# Dynamic evaluation of low-carbon development in China's power industry and the impact of carbon market policies

**DOI:** 10.1016/j.heliyon.2023.e13467

**Published:** 2023-02-03

**Authors:** Hao Liang, Yingying Zeng, Xuchu Jiang, Ying Li

**Affiliations:** Zhongnan University of Economics and Law, Wuhan 430073, China

**Keywords:** Low carbon development, Evaluation index system, Dynamic evaluation, Carbon market policy

## Abstract

At the 75th session of the United Nations General Assembly, China clearly put forward the goals of "carbon peak" in 2030 and "carbon neutrality" in 2060. Achievement of carbon targets. Therefore, the goal of this paper is to analyze the low-carbon development level of China's power industry and study the impact of carbon market policies on the low-carbon development level of the power industry. Based on this, this paper first constructs the low-carbon development evaluation index system of the power industry around the connotation of low-carbon development in the power industry and uses the global principal component analysis model to measure the low-carbon development level of China's power industry. Then, the dynamic change trend and spatial distribution characteristics of the low-carbon development level of China's power industry are analyzed using kernel density estimation and the K-means clustering method. Finally, propensity score matching and difference-in-difference methods are used to analyze the impact of carbon market policies on the low-carbon development level of China's power industry. The results show that, first, the low-carbon development level of China's power industry generally shows an upward trend and a polarized development trend. Second, the low-carbon development level of China's power industry has regional effects and gradient effects. The low-carbon development level of the power industry from high to low is the eastern region, central region and western region. Third, carbon market policies can help improve the low-carbon development level of China's power industry. The research results provide some reference and guidance for the evaluation of the low-carbon development level of China's power industry and the improvement of carbon market policies.

## 1 Introduction

During 1850–2019, human beings emitted approximately 23.9 trillion tons of carbon dioxide. Since the 21st century, the global surface temperature has increased by approximately 0.99 °C compared with that before industrialization [1]. The climate has undergone profound changes, and extreme weather and natural disasters have become more frequent, posing a further threat to human survival and development. In recent years, greenhouse gas emission reduction has gradually become a worldwide issue. Most countries in the world have put forward their own carbon neutrality or net zero emission targets by submitting them to the United Nations, policy declarations or legislation. China, as the largest carbon emitter in the world today, proposed at the 75th session of the United Nations General Assembly in 2020 that carbon dioxide emissions should reach their peak by 2030 and strive to achieve the goal of carbon neutrality by 2060. At the 76th session of the United Nations General Assembly in 2021, China reiterated the above objectives and announced that it would not build new overseas coal-fired power projects.

At present, the carbon emissions of China's power industry account for more than 40% of the total carbon emissions in China, and the installed capacity of coal-fired electricity accounts for nearly 50% [2]. The power generation energy structure is dominated by coal, which still has a high dependence on fossil energy. Fossil energy produces excessive greenhouse gases, which is an important factor causing the current climate problem [3]. As a direct substitute for fossil energy, renewable energy has huge reserves around the world, theoretically exceeding the current energy demand in the world, and making full use of renewable energy can improve energy coverage and security and greatly reduce carbon emissions [4]. At present, renewable energy has been widely used in the power industry. Renewable energy power generation (such as hydropower, solar photovoltaic power generation, wind power, etc.) and energy storage systems are two important means for the low-carbon development of the power industry [5]. In addition, considering the economic benefit and technical difficulty, the power industry has a higher carbon emission reduction efficiency than other industries and has a larger space for emission reduction [6]. Therefore, to achieve the double-carbon goal, China must take the power industry as an important breakthrough and vigorously promote the low-carbon development of the power industry.

The concept of "low carbon" first appeared in the British government's energy white paper [7]. In a narrow sense, "low carbon" refers to lower carbon emissions; broadly speaking, "low carbon" refers to lower carbon emissions, pollution and energy consumption [8]. Currently, an increasing number of countries attach importance to the synergistic effect of carbon emission reduction, that is, the effect of air pollutant emission reduction brought by measures to control carbon dioxide emissions. In addition, in the production process, the emission of carbon dioxide is often accompanied by the emission of pollutants, and the two have homology [9]. Therefore, from the perspective of practical significance, it is more appropriate to adopt the broad concept of low-carbon development; that is, low-carbon development refers to a sustainable development mode characterized by low-carbon emissions, low pollution and low energy consumption, which can improve economic and social benefits.

At present, scholars' research on the low-carbon development of the power industry mainly focuses on the narrow level of "low-carbon", that is, to conduct research around "carbon emissions". This type of research is divided into two categories. One is to study the influencing factors of carbon emissions of the power industry and decompose and identify the factors. The positive driving factors are fossil fuel structure, thermal power generation rate, transmission and distribution loss, net power export, per capita GDP, total population, and per capita living electricity consumption [10]. The negative driving factors include thermal power energy consumption intensity, power structure and power consumption intensity [11]. The second is to study the carbon emission performance or carbon emission efficiency of the power industry. Most of these studies adopt the input–output perspective, mainly using data envelopment analysis (DEA), stochastic frontier analysis (SFA) or the modified versions of these two models (DEA-Malmquist, 3-stage DEA, etc.) for analysis [12]. In a broad sense, low-carbon development involves carbon emissions, pollution emissions, energy consumption, etc. [13], but existing research usually only involves carbon emissions and cannot comprehensively evaluate the low-carbon development level of the power industry. Therefore, it is of great practical significance to build an evaluation index system of the low-carbon development of the power industry from the broad sense of "low-carbon" and analyze the trend and characteristics of the low-carbon development of China's power industry, which provides a certain reference for the evaluation of the low-carbon development level of China's power industry.

To promote the low-carbon development of the power industry, the Chinese government has adopted a series of low-carbon policies - carbon markets, green certificates, green electricity and energy use rights trading. Among them, the carbon market policy started earlier. Scholars’ research on carbon market policy mainly focuses on the impact of carbon market policy on the power industry and analyzes the impact of carbon market policy on the technology, cost, and emissions of the power industry [14]. Considering the correlation and coordination between market-oriented policies, some scholars have discussed the coupling effect and joint mode of the above policies [15]. At present, the literature on the impact of the carbon market focuses on the analysis of the impact of the carbon market on specific aspects of the power industry (such as technology, cost, etc.), but few studies quantitatively analyze the impact of the carbon market on the low-carbon development level of the power industry.

Based on this, this paper first measures the low-carbon development level of the power industry. Then, we analyze the dynamic change trend of the low-carbon development level of the power industry from the three dimensions of the whole country, three major regions and provinces and discuss the spatial distribution characteristics of the low-carbon development level of the power industry. On this basis, the impact of carbon market policies on the low-carbon development level of China's power industry is studied.

In summary, the possible innovations and main contributions of this paper can be summarized into four points. First, studying the low-carbon development level of China's power industry from the broad level of "low-carbon" will help to comprehensively analyze the low-carbon development of the power industry and make up for the fact that only "low-carbon" is usually involved in existing research. Insufficiency in the narrow sense. Second, an evaluation index system including 20 specific indicators of 5 subsystems, including carbon emissions, pollution emissions, energy utilization and consumption, economic benefits and social benefits, has been constructed, making the low-carbon development of the power industry measurable, reportable, and comparable. It is helpful to comprehensively and objectively grasp the low-carbon development level of China's power industry. Third, the global principal component analysis model is used to dynamically measure the low-carbon development level of the power industry, which ensures the integrity, unity and comparability of the system analysis and overcomes the existing problems in some current low-carbon development evaluation index systems of the power industry. The lack of static. Fourth, the impact of carbon market policies on the low-carbon development of the power industry should be more scientifically assessed. Different from the literature, this paper innovatively regards the level of low-carbon development (rather than carbon emissions) as the impact object of carbon market policies, which can more comprehensively and accurately measure the impact of carbon market policies on the low-carbon development of the power industry. It fills a gap in carbon market policy research and provides new ideas and useful thinking for future research.

## 2 Research methods and data preprocessing

**Fig. 1:**
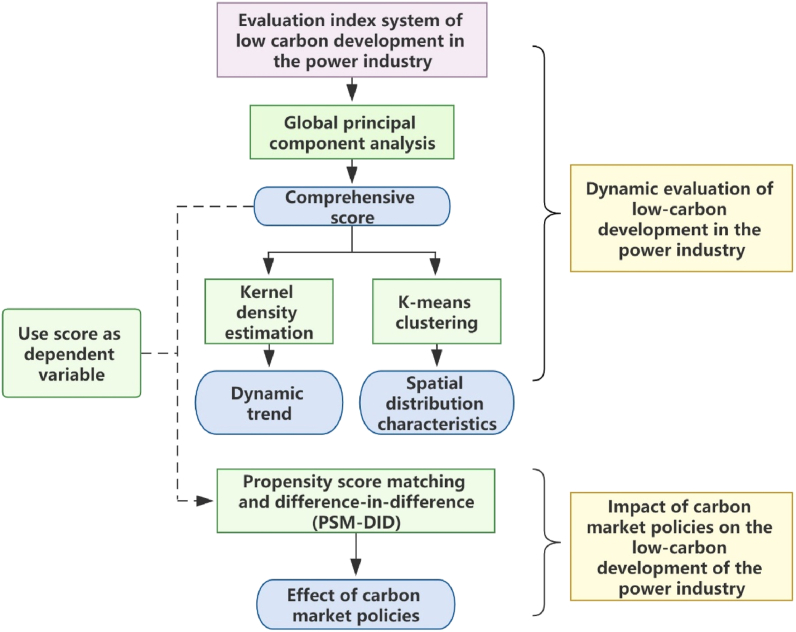
The basic framework of this paper.

This paper first uses the global principal component analysis model to measure the low-carbon development level of the power industry in 30 provinces in China from 2006 to 2019. Then, kernel density estimation is used to analyze the dynamic change trend of the low-carbon development level of the power industry from the two dimensions of the whole country and the three major regions, and the K-means clustering method is used to analyze the spatial distribution characteristics of the low-carbon development level of the power industry. On this basis, the propensity score matching and difference-in-difference methods (PSM-DID) are used to study the impact of carbon market policies on the low-carbon development level of China's power industry (see Fig. 1).

### 2.1 Global principal component analysis

Global principal component analysis is the combination of time series analysis and principal component analysis. The results obtained by global principal component analysis in different periods are comparable, but principal component analysis does not have this advantage. The main idea of this method is to reduce the dimension, remove the correlation of many evaluation indexes and give each evaluation index an objective weight [16].

The main steps of global principal component analysis are as follows:

(1) Establish a three-dimensional time series data table. If the same P indicators are used to describe N regions, one data table ${\left(X_{ij}\right)}_{N\times P}$ can be obtained every year, and there are T data tables in T years. The data tables of each year are arranged from top to bottom in chronological order to form a three-dimensional time series data table, which is marked as ${\left(X_{ij}\right)}_{T\times N\times P}$.
(2) Standardize the normal distribution of the original data to eliminate the influence of dimensions.
(3) The global correlation coefficient matrix is obtained according to the standardized data matrix, and the eigenvalues and eigenvectors are calculated by using the matrix.
(4) Calculate the contribution rate and cumulative contribution rate of global principal components and generally select the global principal components corresponding to the eigenvalues whose cumulative contribution rate is more than 85%.

The eigenvalue distribution obtained in this paper is shown in Table 1. According to the above principles, the first eight global principal components are extracted, and the cumulative variance contribution rate reaches 86.936%.

(5) Taking the contribution rate of global principal components as the weight, the comprehensive score of the low-carbon development of China's power industry is weighted.

**Table 1:** Eigenvalue distribution of the eight global principal components extracted.

The i-th principal component	Eigenvalue		
	Amount to	Contribution rate (%)	Cumulative contribution rate (%)
1	5.473	27.364	27.364
2	4.250	21.520	48.614
3	2.195	10.974	59.588
4	1.614	8.071	67.659
5	1.227	6.134	73.794
6	1.054	5.268	79.062
7	0.808	4.038	83.100
8	0.767	3.836	86.936

### 2.2 Kernel density estimation method

Kernel density estimation is a nonparametric estimation method [17]. From the curve image obtained by kernel density estimation, the distribution position, shape, ductility and other characteristics of random variables can be observed. For random variable X, the specific form of kernel density estimation is Eq. (1):

(1) $$\hat{f}\left(x\right)=\frac{1}{nh}\sum_{i=1}^{n}K\left(\frac{x_{i}-x}{h}\right)$$

In formula (1), n represents the observations, x represents the mean value, h represents the bandwidth and K(*) represents the kernel function. In this paper, the Gaussian kernel density function is used.

### 2.3 K-means clustering method

The main idea of k-means clustering is that all samples can be divided into H classes at first, and H initial clustering centers are selected. Then, the samples are classified according to the principle of minimum distance, and then the clustering centers are iterated continuously until the iteration converges or the clustering centers no longer change. In this process, the clustering situation will be adjusted according to the changes in the clustering centers [18].

### 2.4 PSM-DID model

In the PSM-DID model, the propensity score matching model (PSM) is responsible for matching the individuals in the treatment group with those in the control group, and the difference in difference model (DID) is responsible for identifying the impact of policy shocks. The basic idea of PSM-DID is to set a treatment variable ${treat}_{i}$ and divide samples into a treatment group and a control group. According to the treatment variable ${treat}_{i}$ and the covariate $x_{i}$, the propensity score is estimated, and the samples with the same or close propensity score in the treatment group and the control group are matched. Then, the matched samples are differentiated twice, and the final average treatment effect (ATT) is the policy effect to be studied [19].

To a certain extent, the PSM-DID reduces the dependence on function form setting and alleviates the problem of incorrect setting of function form [20].

The specific form of the DID model adopted in this paper is as follows Eq. (2):

(2) $${CS}_{it}=\beta_{0}+\beta_{1}{treat}_{i}*{post}_{t}+\mu X_{it}+\lambda_{i}+\lambda_{t}+\varepsilon_{it}$$

${CS}_{it}$ Comprehensive score of low-carbon development of the power industry representing province I in year T; $\beta_{0}$ is a regression constant; ${treat}_{i}$ indicates whether the carbon market policy has been implemented. If the province has implemented the policy, then ${treat}_{i}=1$; if the province has not implemented the policy, then ${treat}_{i}=0$; ${post}_{t}$ is a time variable. If the time is before the time of policy implementation, then ${post}_{t}=0$, if the time is after the time of policy implementation, then ${post}_{t}=1$, as most of the carbon market pilots are fully launched in 2014, this paper, like most studies, takes 2014 as the benchmark and defines 2014 as the nonpilot period (${post}_{t}=0$), and 2014 and beyond as the pilot period (${post}_{t}=1$); ${treat}_{i}*{post}_{t}$ is the core explanatory variable, which refers to the interaction term between grouped dummy variables and dummy variables before and after policy implementation, and its coefficient $\beta_{1}$ reflects the net effect of policy implementation; $X_{it}$ represents control variables, including economic development level GDP (regional OPEN capita GDP), industrial structure IS (the added value of secondary industry accounts for the proportion of regional GDP), power consumption intensity ECI (the ratio of power consumption to regional GDP), the number of patent applications granted RD (the number of patents granted by provinces and cities), the degree of opening to the outside world (the ratio of total import and export trade to regional GDP), population size PS (the number of permanent residents at the end of each province) and environmental regulation EGI. $\lambda_{i}$ represents the individual fixed effect; $\lambda_{t}$ represents the time fixed effect; $\varepsilon_{it}$ is a random interference term.

Due to the lack of a comprehensive score of the low-carbon development of the power industry in some areas in 2017, this paper excludes the data in 2017 when using the PSM-DID model; that is, the panel data of 30 provinces in China (excluding Hong Kong, Macau, Taiwan Province and Tibet due to lack of data) from 2006 to 2019 (excluding 2017) are adopted.

### 2.5 Data preprocessing

#### 2.5.1 Data sources

Considering the research purpose, timeliness and availability of data, this paper selects sample data from 30 provinces in China (including municipalities and autonomous regions, Hong Kong, Macau, Taiwan Province and Tibet) from 2006 to 2019 for research. Among them, carbon emission data come from the China Carbon Accounting Database (CEADs), and other data come from the National Bureau of Statistics, China Statistical Yearbook (2006–2019), China Electric Power Yearbook (2006–2019), Compilation of Electric Power Industry Statistics (2006–2019), and China Industrial Statistical Yearbook (2006–2019). The yearbook lacks data from 2017 to 2018, the China Economic Census Yearbook (2018), the China Energy Statistics Yearbook (2006–2019), the China Labor Statistics Yearbook (2006–2019), the China Environmental Statistics Yearbook (2006–2015), the China Ecological Environment Statistical Annual Report (2016–2019) and provincial statistical yearbooks.

#### 2.5.2 Processing of missing values

The evaluation index system of the low-carbon development of the power industry designed in this paper has 20 indicators, covering 30 provinces in China from 2006 to 2019 (the reasons for the missing areas are as mentioned above). It is planned to collect 8400 sample data points (20*14*30), 23 data points are missing, and 8377 sample data points are obtained. The missing data are shown in Table 2.

**Table 2:** Missing data.

Index name	Year and province
Standard coal consumption for power supply	Chongqing from 2008 to 2011
Standard coal consumption for power generation	Chongqing from 2008 to 2011
Return on sales	Inner Mongolia in 2017, Heilongjiang in 2017
Asset-liability ratio	Inner Mongolia in 2017, Guangdong in 2017, Guangxi in 2017 and Guizhou in 2017
Cost per hundred yuan of operating income	2017 Inner Mongolia, 2017 Heilongjiang, 2017 Fujian, 2017 Shandong, 2017 Guangxi, 2017 Guizhou, 2017 Yunnan
Main business income realized per hundred yuan of assets	Inner Mongolia in 2017, Heilongjiang in 2017

Among the missing data, the data of standard coal consumption for power supply and standard coal consumption for power generation in the Chongqing power industry from 2008 to 2011 are approximately linear and show a monotonic decreasing trend, which is supplemented by linear interpolation. As there is no obvious change trend or rule in the remaining data, the deletion method is adopted. For example, in 2017, the index data of cost per hundred yuan of operating income of the Yunnan power industry are missing, so the Yunnan power industry will not participate in the scoring in 2017.

#### 2.5.3 Uniform and dimensionless treatment

There are both positive and negative indicators in the evaluation index system of the low-carbon development of the power industry, which will affect the results of the global principal component analysis. The processing method of index unification is to translate the reverse index, $x_{j}^{*}=M_{j}-x_{j}$, where $M_{j}$ indicates the largest index value in the j-th item of n evaluation objects. After unification, all the index data are normalized by a normal distribution.

## 3 Construction of an evaluation index system for the low-carbon development of the power industry

Following the five principles of embodying core values, system comprehensiveness, concise scientific, dynamics and operability, and centering on the connotation and concept of "low-carbon development", this paper constructs the evaluation index system of the low-carbon development of China's power industry from five aspects: carbon emissions, pollution emissions, energy utilization and consumption, economic benefits and social benefits (Table 3). The details are as follows:

(1) Carbon emissions. The criterion layer of carbon emissions reflects the carbon emissions of the power industry and is one of the most important manifestations of the low-carbon development of the power industry. The criterion layer consists of two indicators, which measure the carbon emissions of the power industry from two dimensions: total amount and efficiency. Carbon emissions can directly reflect the carbon emissions of the power industry, and the carbon intensity of electric power reflects the carbon emission efficiency of the power industry.
(2) Pollution discharge. The criterion layer of pollution reflects the air pollutant emissions in the power industry. Promoting coordinated emission reduction of air pollutants and carbon dioxide is an inherent requirement and an important part of low-carbon development. Sulfur dioxide, nitrogen oxides and smoke dust are the main air pollutants produced in the production process of the power industry, and their emission rates can measure the pollution emission efficiency of the power industry.
(3) Energy utilization and consumption. The criterion layer of energy utilization and consumption reflects the energy utilization efficiency and energy structure of the power industry, while the essence of low carbon is to improve energy utilization efficiency and clean energy structure, which is an important reflection of the essence of low carbon [21]. Standard coal consumption for power supply, standard coal consumption for power generation, application rate of energy storage technology and line loss rate measure the energy utilization efficiency of the power industry, while the proportion of low-carbon energy installed capacity, the proportion of electricity generated by low-carbon energy units and the application degree of low-carbon transmission technology measure the energy structure of the power industry.
(4) Economic benefits. The criterion layer of economic benefit reflects the economic achievements and efficiency of the power industry in the process of low-carbon development. The index setting refers to the industrial statistical report system (2021) promulgated by the National Bureau of Statistics and takes into account the particularity of the power industry. Electricity sales, utilization hours of power generation equipment, return on sales, asset-liability ratio, cost per hundred yuan of operating income and main business income per hundred yuan of assets can fully reflect the economic benefits of the power industry.
(5) Social benefits. The criterion layer of social benefit reflects the social contribution made by the power industry in the process of low-carbon development. Among the social contributions, the most prominent is employment, which is the foundation and premise of social and economic development. Employment population and average wage can measure the social benefits of the power industry.

**Table 3:** Evaluation index system of low carbon development in China's power industry.

Target layer	Criterion layer	Index layer	Meaning and unit of indicators	Index direction
Evaluation index system of low-carbon development in China's power industry	Carbon emission	Carbon emission	Total carbon emission of power industry (tons)	minus
		Carbon intensity	${CO}_{2}$ emission per unit power generation (g/kWh)	minus
	Pollution emission	Sulfur dioxide emission rate	${SO}_{2}$ emission per unit power generation (g/kWh)	minus
		Nitrogen oxide emission rate	${NO}_{x}$ emission per unit power generation (g/kWh)	minus
		Smoke dust emission rate	Smoke and dust emission caused by unit power generation (g/kWh)	minus
	Energy utilization and consumption	Standard coal consumption for power supply	Average standard coal consumption per 1 kWh of electricity supplied to the outside (g/kWh)	minus
		Standard coal consumption for power generation	Average standard coal consumption per 1 kWh of electricity (g/kWh)	minus
		Application rate of energy storage technology	Proportion of the capacity of energy storage power stations and devices to the total installed capacity of the system (%)	straight
		Ratio of line loss	Percentage of line loss load to power supply load (%)	minus
		Proportion of low-carbon energy installed capacity	Proportion of installed capacity of low-carbon energy power generation to total installed capacity of the system (%)	straight
		Proportion of electricity generated by low-carbon energy units	Proportion of electricity generated by low-carbon energy units in total electricity generation (%)	straight
		Application degree of low-carbon transmission technology	Application degree of low-carbon transmission and distribution technologies such as UHV transmission and hvdc light (%)	straight
	Economic benefits	Electricity sales	Electricity quantity sold to users by electricity industry (100 million kWh)	straight
		Utilization hours of power generation equipment	Operating hours (hours) of the average capacity of power generation equipment in a certain period under the condition of full load operation	straight
		Return on sales	Ratio of total profit to operating income (%)	straight
		Asset-liability ratio	Ratio of total liabilities to total assets (%)	minus
		Cost per hundred yuan of operating income	Operating cost per operating income of 100 yuan (RMB)	minus
		Main business income realized per hundred yuan of assets	Main business income realized per asset of 100 yuan (RMB)	straight
	Social benefits	Employment population	Number of employees in the power industry at the end of the year (people)	straight
		Average wage	Average wage of employees in the power industry (RMB)	straight

The evaluation index system of the low-carbon development of China's power industry is shown in Table 3.

## 4 Analysis of dynamic evaluation results

### 4.1 KMO test and Bartlett spherical test

Before the global principal component analysis, the sample data need the KMO test and the Bartlett spherical test. As shown in Table 4, the statistical value of the KMO test is 0.720, which is greater than 0.7. The approximate chi-square value of the Bartlett spherical test is 8213.987, and the P value is less than 0.01, which indicates that the correlation between variables is strong, and the sample data are suitable for global principal component analysis.

**Table 4:** KMO test and Bartlett spherical test results.

Research sample	Kaiser-Meyer -Olkin test	Bartlett spherical test	
		Approximate chi-square value	P value
Sample of low-carbon development level of power industry in 30 provinces and cities in China from 2006 to 2019	0.720	8213.987	0.000

### 4.2 Analysis of the low-carbon development trend of China's power industry

**Fig. 2:**
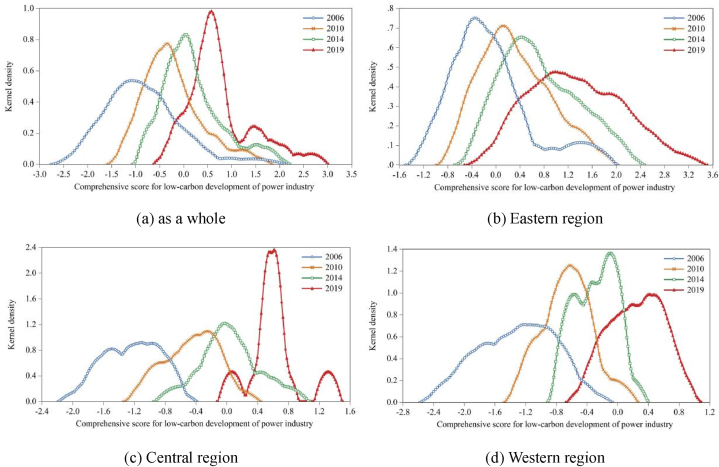
Distribution of the kernel density of the comprehensive score of the low-carbon development of the power industry in the whole and three regions, east, central and west.

The comprehensive score and rankings of the low-carbon development of the power industry in 30 provinces of China from 2006 to 2019 are shown in Table 5 and Table 6. To analyze the dynamic trend of the low-carbon development of the power industry in China more systematically and comprehensively, the distribution charts of the kernel density of the comprehensive score of the low-carbon development of the power industry in the whole region and the three major regions of eastern, central and western China are drawn, and the curves of kernel density in 2006, 2010, 2014 and 2019 are given in Fig. 2. The following will discuss the low-carbon development trend of the power industry from three levels: the whole, three regions and provinces.

**Table 5:** Comprehensive score of the low-carbon development of the power industry in 30 provinces of China from 2006 to 2019.

Region	2006	2007	2008	2009	2010	2011	2012	2013	2014	2015	2016	2017	2018	2019
Beijing	1.4103	1.3584	1.3281	1.3354	1.3712	1.4087	1.5235	1.5470	1.7586	2.3097	2.3570	2.5380	2.5162	2.6470
Tianjin	0.0218	0.1321	0.0247	0.0645	0.4389	0.3028	0.3869	0.4907	0.6133	0.8319	1.4211	1.8204	1.8570	1.9203
Hebei	−0.7392	−0.4783	−0.4291	−0.1924	−0.0395	−0.0311	0.0334	0.1977	0.2893	0.3529	0.5260	0.7906	0.7867	0.8858
Shanxi	−0.9455	−0.6415	−0.6212	−0.4793	−0.3394	−0.3848	−0.2669	−0.0172	−0.0173	0.0619	0.3545	0.5111	0.4616	0.5798
Inner Mongolia	−0.9602	−0.7373	−0.7510	−0.5734	−0.4195	−0.3860	−0.3281	−0.1940	−0.2149	−0.0856	0.0315		0.4298	0.6039
Liaoning	−0.8604	−0.6690	−0.6076	−0.6035	−0.3257	−0.3399	−0.0988	−0.0528	0.0465	0.1798	0.2824	0.3102	0.2777	0.3725
Jilin	−1.7727	−1.2427	−1.0362	−0.9351	−0.8714	−0.6213	−0.3991	−0.2955	−0.1669	−0.0165	0.1545	0.2618	0.3852	0.4094
Amur	−1.5669	−1.3661	−1.3228	−1.2636	−0.9302	−0.8546	−0.7846	−0.7667	−0.5795	−0.4702	−0.2748		0.0155	0.0676
Shanghai	0.4652	0.6396	0.5504	0.7240	1.0336	1.1970	1.3629	1.5135	1.5767	1.6583	1.7872	1.8261	1.8678	2.1267
Jiangsu	−0.3092	−0.1577	−0.0252	0.1566	0.3036	0.4084	0.5923	0.7666	0.9081	1.1011	1.3407	1.4609	1.5720	1.6311
Zhejiang	−0.0072	0.1506	0.2022	0.3175	0.6107	0.7359	0.8709	1.0007	1.2116	1.2708	1.4771	1.5403	1.5280	1.5178
Anhui	−0.9282	−0.6713	−0.3445	−0.1724	0.0449	−0.0088	0.2513	0.5545	0.7176	0.9143	0.9878	1.0729	1.5342	1.3150
Fujian	−0.3979	−0.1903	−0.1652	−0.0816	0.0193	0.2338	0.2671	0.3490	0.4333	0.4432	0.4575		0.6868	0.7637
Jiangxi	−1.4365	−1.0571	−0.9041	−0.5639	−0.3400	−0.3713	−0.2690	−0.1280	0.0124	0.2043	0.4409	0.4511	0.6138	0.6385
Shandong	−0.5883	−0.4668	−0.3584	−0.2503	−0.1595	−0.0850	0.0121	0.1523	0.1609	0.3816	0.4802		0.7863	0.8166
Henan	−1.2152	−0.8150	−0.6763	−0.4696	−0.2311	−0.0845	0.0175	0.1270	0.2090	0.2434	0.3617	0.3679	0.4322	0.5744
Hubei	−0.8031	−0.4516	−0.4284	−0.2467	−0.2333	−0.2007	−0.0469	0.1233	0.3881	0.4648	0.5339	0.6465	0.7296	0.7566
Hunan	−1.4681	−1.2687	−1.2370	−0.9176	−0.7330	−0.5754	−0.4662	−0.2798	0.0209	0.0159	0.0469	0.2062	0.3528	0.5361
Guangdong	0.0270	0.1844	0.1458	0.3168	0.5053	0.6301	0.7053	0.7197	0.7730	0.9241	1.0837		1.2682	1.3906
Guangxi	−1.0551	−0.9344	−0.7426	−0.5864	−0.4537	−0.4299	−0.2893	−0.2004	−0.1004	−0.0355	−0.0579		0.1058	0.2619
Hainan	−0.2479	−0.2161	−0.3437	−0.3802	−0.2558	−0.1285	−0.0161	0.1113	0.1640	0.2539	0.3203	0.1771	0.3530	0.5336
Chongqing	−1.6553	−1.1429	−1.0004	−0.9452	−0.6804	−0.6058	−0.6025	−0.3700	−0.1461	0.0096	0.2687	0.3052	0.3804	0.4933
Sichuan	−1.9059	−1.6201	−1.5202	−1.2602	−1.0386	−0.8579	−0.6843	−0.7495	−0.5367	−0.4916	−0.4705	−0.4075	−0.3083	−0.1262
Guizhou	−1.0368	−1.1174	−0.6978	−0.6667	−0.5895	−0.7132	−0.5905	−0.6364	−0.6086	−0.4444	−0.2581		−0.1785	0.0511
Yunnan	−1.0580	−0.9704	−0.9836	−0.7401	−0.5928	−0.6896	−0.6920	−0.6039	−0.5499	−0.6153	−0.6495		−0.4376	−0.2678
Shaanxi	−1.3079	−0.9587	−0.9610	−0.6968	−0.5440	−0.4477	−0.4036	−0.2095	−0.1117	0.0108	0.2052	0.5375	0.5645	0.6922
Gansu	−0.8380	−0.6224	−0.6092	−0.5559	−0.5585	−0.5604	−0.4732	−0.3926	−0.1809	−0.2134	−0.1801	0.0939	0.0928	0.3506
Qinghai	−1.5653	−1.2216	−0.8064	−0.9521	−0.9264	−0.8176	−0.6938	−0.6245	−0.5796	−0.7161	−0.4268	−0.1829	−0.1637	−0.0713
Ningxia	−0.6198	−0.4506	−0.6254	−0.3069	−0.0924	−0.0989	−0.0298	−0.0261	0.0957	0.4156	0.4132	0.4844	0.5988	0.6289
Xinjiang	−2.0214	−1.7392	−0.9203	−1.5262	−1.1110	−0.8018	−0.5467	−0.2605	−0.1399	−0.0634	−0.0158	0.5738	0.2201	0.4191
Average value	−0.8462	−0.6247	−0.5289	−0.4150	−0.2379	−0.1726	−0.0553	0.0615	0.1815	0.2965	0.4333	0.6993	0.6443	0.7506

Note: If the indicator data of a region are missing in a certain year, the region will not participate in the scoring in the year with missing data, and the above table is blank.

**Table 6:** Ranking of the comprehensive score of the low-carbon development of the power industry in 30 provinces of China from 2006 to 2019.

Region	2006	2007	2008	2009	2010	2011	2012	2013	2014	2015	2016	2018	2019
Beijing	1	1	1	1	1	1	1	1	1	1	1	1	1
Tianjin	4	5	5	6	5	6	6	7	7	7	4	3	3
Hebei	11	12	12	9	9	9	9	9	10	12	9	8	8
Shanxi	16	14	15	15	16	17	16	14	18	17	15	15	16
Inner Mongolia	17	17	20	18	18	18	19	18	25	24	22	17	15
Liaoning	14	15	13	20	15	15	15	16	15	16	17	22	23
Jilin	28	26	27	25	26	24	20	23	23	21	20	18	22
Amur	26	28	29	29	28	29	30	30	28	27	27	26	26
Shanghai	2	2	2	2	2	2	2	2	2	2	2	2	2
Jiangsu	7	6	6	5	6	5	5	4	4	4	5	4	4
Zhejiang	5	4	3	3	3	3	3	3	3	3	3	6	5
Anhui	15	16	9	8	7	8	8	6	6	6	7	5	7
Fujian	8	7	7	7	8	7	7	8	8	9	11	11	10
Jiangxi	23	22	22	17	17	16	17	17	17	15	12	12	13
Shandong	9	11	10	11	11	11	11	10	13	11	10	9	9
Henan	21	18	17	14	12	10	10	11	11	14	14	16	17
Hubei	12	10	11	10	13	14	14	12	9	8	8	10	11
Hunan	24	27	28	24	25	22	22	22	16	18	21	21	18
Guangdong	3	3	4	4	4	4	4	5	5	5	6	7	6
Guangxi	19	19	19	19	19	19	18	19	19	22	24	24	25
Hainan	6	8	8	13	14	13	12	13	12	13	16	20	19
Chongqing	27	24	26	26	24	23	26	24	22	20	18	19	20
Sichuan	29	29	30	28	29	30	27	29	26	28	29	29	29
Guizhou	18	23	18	21	22	26	25	28	30	26	26	28	27
Yunnan	20	21	25	23	23	25	28	26	27	29	30	30	30
Shaanxi	22	20	24	22	20	20	21	20	20	19	19	14	12
Gansu	13	13	14	16	21	21	23	25	24	25	25	25	24
Qinghai	25	25	21	27	27	28	29	27	29	30	28	27	28
Ningxia	10	9	16	12	10	12	13	15	14	10	13	13	14
Xinjiang	30	30	23	30	30	27	24	21	21	23	23	23	21

Note: Due to the lack of a comprehensive score of the low-carbon development of the power industry in some areas in 2017, it is impossible to rank all 30 provinces, so it cannot be compared with other years' rankings. The ranking is of little practical significance, so it will not be ranked in 2017.

#### 4.2.1 Overall trend

By observing the comprehensive score of low-carbon development of the power industry in 30 provinces of China from 2006 to 2019 (Table 5), we can see that the overall level of low-carbon development of the power industry in China is constantly improving and showing an upward trend. The average comprehensive score of low-carbon development in the power industry rose from −0.8462 in 2006 to 0.7506 in 2019, an increase of 1.5968. According to the following kernel density diagram for further discussion, Fig. 2(a) shows the dynamic trend of the low-carbon development of China's power industry from the overall level and shows the following characteristics:

(1) From the distribution position, the peak center and the change range of the kernel density distribution curve show a right shift trend, especially the right shift trend of the left starting point, which shows that the low-carbon development level of the power industry in 30 provinces in China is on the rise as a whole and further confirms the above conclusion from observing the comprehensive score.
(2) From the distribution pattern, the overall performance of the kernel density distribution curve is that the height of the main peak is constantly rising and the width of the curve is decreasing, which means that the absolute difference in the low-carbon development level of the power industry in 30 provinces of China is decreasing. Therefore, from the overall point of view, the absolute gap in the low-carbon development level of the power industry in each province is further narrowed.
(3) From the point of view of distribution ductility, there is a right tailing phenomenon in the kernel density distribution curve, and the distribution ductility shows a changing process of first converging and then widening. This shows that the gap between the provinces with higher low-carbon development levels and the provinces with lower-carbon development levels in the power industry first narrowed and then widened. The provinces with higher low-carbon development levels in the power industry have ushered in a new wave of development momentum in recent years, which may be influenced by relevant low-carbon pilot policies, such as the carbon market policy.
(4) From the polarization phenomenon, the kernel density distribution curve experienced a process from the initial single peak state to the "one main side" double peak state during the observation period. The bimodal state consists of a main peak and a right peak. The right peak began to appear in 2014 and became more prominent with the passage of time, forming an obvious right peak in 2019. This shows that the low-carbon development level of the power industry in 30 provinces of China presents a polarization trend. In addition, the peak value of the side peak is obviously lower than that of the main peak, indicating that there is a gradient effect.

In summary, the overall level of low-carbon development in China's power industry is constantly improving, and the absolute difference is also showing a certain narrowing trend. However, the polarization trend is becoming increasingly obvious, reflecting the characteristics of decentralized regional agglomeration.

#### 4.2.2 Three regional trends

Fig. 2(b–d) show the dynamic trends of the low-carbon development of the power industry in the eastern, central and western regions, respectively. The specific descriptions are as follows:

(1) From the distribution position, the peak center and change range of the kernel density distribution curve in the eastern, central and western regions all show a right shift trend, which indicates that the low-carbon development level of the power industry in the three regions is generally on the rise. In addition, from the distance of the location movement, the moving distance of the kernel density curve to the right in the central and western regions is larger than that in the eastern region, which indicates that the low-carbon development level of the power industry in the central and western regions is progressing faster, and the gap with the eastern region is further narrowed.
(2) From the distribution pattern, the distribution curve of kernel density in the eastern region generally shows that the height of the main peak shows a downward trend and the curve gradually widens, which indicates that the absolute difference in the low-carbon development level of the power industry in the eastern region shows an expanding trend. Overall, the distribution curve of kernel density in the central region shows that the height of the main peak is rising and the curve is gradually narrowing, and the height of the main peak in 2019 is much higher than that in 2006, 2010 and 2014, which indicates that the absolute difference in the low-carbon development level of the power industry in the central region is shrinking and the degree of agglomeration is highly expanding. In general, the distribution curve of kernel density in the western region shows that the height of the main peak increases first and then decreases, and the width of the curve decreases first and then expands, which indicates that the absolute difference in the low-carbon development level of the power industry in the western region shows a trend of first narrowing and then expanding, but overall, the absolute difference shows a weak narrowing trend.
(3) From the perspective of distribution extensibility, there is a right tailing phenomenon in the eastern region, but there is no obvious tailing phenomenon in the central and western regions. On the whole, the extensibility of the eastern region converges first and then widens to the right, which indicates that the gap between the provinces with higher low-carbon development levels and the provinces with lower levels of power industry in the eastern region narrows first and then widens, which is similar to the overall development trend. The extensibility of the central region has experienced the process of "substantial convergence-slight widening-slight convergence", but on the whole, it is in a state of substantial convergence, which indicates that the gap between the provinces with higher and lower levels of low-carbon development in the power industry in the central region has narrowed considerably, and the low-carbon development level in the region has become more balanced. The extensiveness of the western region has experienced a process of "large convergence-small convergence-widening", which shows a convergent trend overall, which means that the gap between the provinces with higher low-carbon development levels and the provinces with lower levels of power industry in the western region has narrowed overall.
(4) From the polarization phenomenon, the eastern region was initially in a double peak state, but the right peak was not obvious. The kernel density curve in 2010 showed that it had evolved into a single peak state, and then it remained in a single peak state until 2019, indicating that the low-carbon development level of the power industry in the eastern region has a multipolarization development trend. The peak state of the central region has experienced the changing process of "double peaks-inconspicuous double peaks-single peak-triple peaks", which shows that with the passage of time, the low-carbon development level of the power industry in the central region has gradually evolved from a single polarization trend to a triple polarization trend, and the left peak and the right peak of the triple peak state are obviously lower than the main peak, which means that there is a significant gradient effect in this region. The peak state of the western region has experienced the changing process of "single peak-three peaks-inconspicuous double peaks", which indicates that the low-carbon development level of the power industry in the western region presents a multipolarization development trend at the initial stage and then gradually develops toward dual polarization or single polarization.

In summary, the low-carbon development level of the eastern, central and western power industries is on the rise. The eastern region maintains the characteristics of single agglomeration, but the degree of agglomeration has decreased. The central region is characterized by decentralized agglomeration, and the level of agglomeration is greatly improved. The concentration degree of the western region is relatively stable, and the concentration level has slightly increased.

#### 4.2.3 Provincial trends

**Fig. 3:**
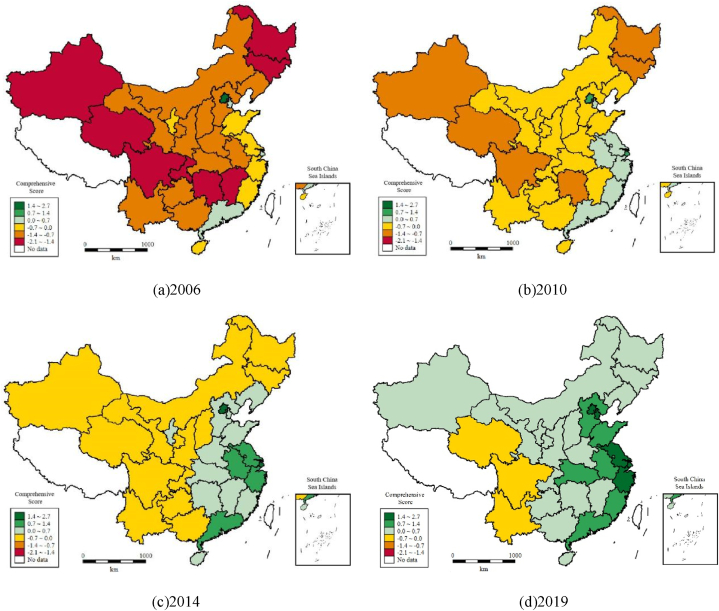
Evolution trend of the low-carbon development level of the power industry in 30 provinces of China from 2006 to 2019.

From the observation of Fig. 3, combined with the comprehensive score and ranking, the change in the low-carbon development level of the power industry in each province has different characteristics, but it shows a fluctuating upward trend overall.

Judging from the comprehensive score, Beijing, Tianjin, Shanghai and Guangdong all have positive comprehensive scores over the years, and their power industry has always maintained a high level of low-carbon development. These provinces and cities are all developed areas, and their economic development, scientific and technological level, openness and talent reserve rank among the top in the country. Sichuan, Yunnan and Qinghai, which all have negative comprehensive scores over the years, belong to the western region. Although the low-carbon development level of their power industry is on the rise in general, compared with other provinces, the low-carbon development level is relatively backward. There are 23 provinces that have changed from negative values to positive values, and the process of transformation varies from province to province. Among them, Jiangsu, Zhejiang, Anhui, and Fujian turned positive in five years (2006–2010), while Shanxi, Inner Mongolia, Jilin, Heilongjiang, Guangxi, Guizhou, Shaanxi, Gansu, and Xinjiang only turned positive in the last five years (2015–2019).

From the ranking point of view, Beijing has been ranked first all the years, and its low-carbon development level of the power industry has always maintained a leading position. The provinces with the greatest progress are Jiangxi and Shaanxi, ranking 10 places ahead, which shows that compared with other provinces, the low-carbon development level of the power industry in these two provinces has made rapid progress and made great progress. The province with the largest regression is Hainan, which dropped from 6th place in 2006 to 19th place in 2019, with a decline of 13 places, indicating that the progress of the low-carbon development level of the power industry in this province is lagging behind, and more effective low-carbon development measures need to be taken.

### 4.3 Cluster analysis of low carbon development in China's power industry

The dynamic trend of low-carbon development in China's power industry has been analyzed at multiple levels. To further explore the spatial distribution characteristics of low-carbon development in China's power industry, this paper uses the k-means clustering method to divide the low-carbon development level of the power industry in each province into four echelons according to the clustering results, as shown in Table 7. Overall, the composition of the echelon is relatively stable, and the echelons to which most provinces belong have not changed.

**Table 7:** K-means clustering echelon division of low-carbon development evaluation of the power industry in 30 provinces of China.

	2006	2010	2014	2019
The first echelon	Beijing	Beijing, Shanghai	Beijing, Shanghai	Beijing
The second echelon	Tianjin, Shanghai, Jiangsu, Zhejiang, Fujian, Guangdong, Hainan	Tianjin, Jiangsu, Zhejiang, Guangdong	Tianjin, Jiangsu, Zhejiang, Anhui, Guangdong	Tianjin, Shanghai, Jiangsu, Zhejiang, Anhui, Guangdong
The third echelon	Hebei, Shanxi, Inner Mongolia, Liaoning, Anhui, Shandong, Henan, Hubei, Guangxi, Guizhou, Yunnan, Gansu, Ningxia	Hebei, Shanxi, Inner Mongolia, Liaoning, Anhui, Fujian, Jiangxi, Shandong, Henan, Hubei, Guangxi, Hainan, Guizhou, Yunnan, Shaanxi, Gansu, Ningxia	Hebei, Shanxi, Inner Mongolia, Liaoning, Jilin, Fujian, Jiangxi, Shandong, Henan, Hubei, Hunan, Guangxi, Hainan, Chongqing, Shaanxi, Gansu, Ningxia, Xinjiang	Hebei, Shanxi, Inner Mongolia, Liaoning, Jilin, Fujian, Jiangxi, Shandong, Henan, Hubei, Hunan, Guangxi, Hainan, Chongqing, Shaanxi, Gansu, Ningxia, Xinjiang
The fourth echelon	Jilin, Heilongjiang, Jiangxi, Hunan, Chongqing, Sichuan, Shaanxi, Qinghai, Xinjiang	Jilin, Heilongjiang, Hunan, Chongqing, Sichuan, Qinghai, Xinjiang	Heilongjiang, Sichuan, Guizhou, Yunnan, Qinghai	Heilongjiang, Sichuan, Guizhou, Yunnan, Qinghai

The first echelon is Beijing and Shanghai, of which Beijing has been in the first echelon, and Shanghai mainly lies between the first and second echelons. These two cities belong to China's super first-tier cities, with abundant financial resources, material resources and manpower, excellent resource endowment, a high level of environmental governance, and excellent performance in the low-carbon development of the power industry. In addition, they are pilot cities for carbon market policies supported by relevant environmental policies.

The second echelon is mainly composed of Tianjin, Jiangsu, Zhejiang, Anhui and Guangdong. Among these five cities, four belong to the eastern region and one belongs to the central region, among which Tianjin and Guangdong are the pilot provinces and cities of carbon market policy. These provinces and cities have the highest level of economic development in the country, high resource utilization efficiency, and great achievements in the low-carbon development of the power industry. The changes in the second echelon mainly come from Fujian, Hainan and Anhui provinces, among which Fujian and Hainan fell from the second echelon to the third echelon in 2010, and Anhui climbed from the third echelon to the second echelon in 2014.

The number of provinces and cities in the third echelon is the largest, and with the passage of time, it shows an upward trend, increasing from 13 in 2006 to 18 in 2019, accounting for more than 50% of the national total. Take the third echelon in 2014 and 2019 as an example. Among these 18 provinces and cities, the eastern region accounts for 5, the central region accounts for 7, and the western region accounts for 6. The regional distribution of this echelon is relatively uniform. The economic development level and endowment conditions of the provinces and cities in this echelon are uneven, among which the provinces with higher economic development levels are Henan, Hubei, Fujian and Hunan, while the provinces with relatively backward economic development are Jilin, Gansu, Ningxia and Xinjiang. The changes in the third echelon mainly come from nine provinces and cities: Jilin, Fujian, Hainan, Anhui, Hunan, Yunnan, Shaanxi, Xinjiang and Chongqing. With the passage of time, Anhui and Yunnan left the third echelon, and Jilin, Fujian, Hainan, Hunan, Shaanxi, Xinjiang and Chongqing joined the third echelon. The increase in the third echelon mainly comes from the fourth echelon, which indicates that the provinces with a low-carbon development level of the power industry have an upward trend of agglomeration.

The fourth echelon is mainly composed of five provinces: Heilongjiang, Sichuan, Guizhou, Yunnan and Qinghai, with one in the central region and four in the western region. These provinces are geographically remote and located in the interior of China, with a low degree of openness to the outside world, a relatively underdeveloped level of science and technology, a low level of low-carbon development of the power industry, and relatively weak development. Except for Sichuan, the economic level of other provinces is relatively backward, and the efficiency of resource utilization needs to be further improved. It is worth noting that the number of provinces constituting this echelon is gradually decreasing, and some provinces have achieved a leap of echelon, which has the potential to further improve the level of low-carbon development.

In summary, the provinces and cities in the eastern region are mainly distributed in the first and second echelons, the provinces and cities in the central region are mainly distributed in the second and third echelons, and the provinces and cities in the western region are mainly distributed in the third and fourth echelons. There are gradient effects and regional effects in the spatial distribution of the low-carbon development level of China's power industry, and generally, there is a trend that lower echelons converge to higher echelons.

## 5 Analysis of the influence of carbon market policies on the low-carbon development of China's power industry

The temporal and spatial differentiation of the low-carbon development of the power industry is caused by many factors, including environmental policy, resource endowment, economy and society, technical level and so on. Considering the actual needs of China's environmental policy improvement and the abundant research results on resource endowment, economy, society, technology level and other influencing factors, this paper mainly studies the impact of environmental policy on the low-carbon development of the power industry. Among environmental policies, carbon market policy is an important market-oriented emission reduction tool to achieve China's peak carbon dioxide emission and carbon neutrality goals. Compared with developed countries and regions, China's carbon market construction is at the initial stage of development, and related issues need to be improved, which is of great research value.

At the same time, through the above global principal component analysis and cluster analysis, it can be found that the power industry in the pilot provinces and cities of carbon market policy has a high level of low-carbon development, with outstanding performance, such as Beijing, Tianjin, Shanghai and Guangdong, which are located in the first and second echelons, respectively. In addition, according to the cluster analysis table (Table 7), Chongqing (one of the pilot cities) climbed from the fourth echelon to the third echelon in 2014, realizing the leap of the echelon. The above results show that carbon market policy may play a positive role in the low-carbon development of the power industry in pilot cities. To further explore the impact of carbon market policy on the low-carbon development of the power industry, this paper uses the PSM-DID model for analysis.

### 5.1 Parallel trend test and dynamic effect analysis of the policy

The premise of using the DID model is to meet the parallel trend condition, that is, the treatment group and the control group have the same change trend before the implementation of the carbon market policy, and there is no systematic difference. In this paper, the event study method is used for analysis. The event study method can not only test the parallel trend hypothesis but also study the dynamic effect of carbon market policy. According to the principle of the event study method, the interaction term of the year dummy variable and the policy dummy variable is used as the independent variable of the comprehensive score of the low-carbon development of the power industry to construct the model. The specific form is as follows Eq. (3):

(3) $${CS}_{it}=\beta_{0}+\sum_{t=2006}^{2019}\beta_{t}D_{it}+\mu X_{it}+\lambda_{i}+\lambda_{t}+\varepsilon_{it}$$

$D_{it}$ is the dummy variable of province I in year T (the interaction term of the year dummy variable and policy dummy variable); when province I is in year T, $D_{it}=1$; otherwise, it is 0. When the coefficient $\beta_{t}$ ($\beta_{2006}-\beta_{2012}$) of the dummy variable $D_{it}$ before the implementation of the policy is not significant, it means that the comprehensive score of low-carbon development of the power industry of the treatment group and the control group have the same change trend before the implementation of the carbon market policy and pass the parallel trend test. It should be noted that the base year is set to 2013, the year before the implementation of the policy, so there is no independent variable $D_{2013}$ in the model. In addition, as mentioned above, due to the lack of a comprehensive score in some areas in 2017, the panel data of 2006–2019 (excluding 2017) are used when using the DID model, so the independent variable $D_{2017}$ does not exist.

The estimated results are shown in Table 8. The coefficients $\beta_{t}$ of the dummy variables $D_{it}$ from 2006 to 2012 are negative and not significant, indicating that before the implementation of the carbon market policy, the change trend of the comprehensive score of the low-carbon development of the power industry in the treatment group and the control group is consistent, meeting the parallel trend hypothesis. In addition, from the perspective of policy dynamic effects, in the year when the carbon market policy was implemented, that is, in 2014, the coefficient turned from negative to positive, but it has not yet passed the significance test. However, in the first, second, fourth and fifth years after the implementation of the policy, the coefficient is always positive and passes the significance tests of 10%, 5%, 10% and 5%, respectively, indicating that there is a certain lag in the policy effect of the carbon market. From the angle of coefficient value, the coefficient shows an increasing trend with the passage of time after the implementation of carbon market policy, which indicates that the positive impact of carbon market policy on the low-carbon development of the power industry is gradually expanding, and the policy effect of the carbon market is sustainable and growing.

**Table 8:** Parallel trend test.

The year of the dummy variable $D_{it}$	Test result
2006 (8 years ago)	−0.083 （0.162）
2007 (7 years ago)	−0.058 （0.128）
2008 (6 years ago)	−0.153 （0.125）
2009 (5 years ago)	−0.124 （0.096）
2010 (4 years ago)	−0.078 （0.079）
2011 (3 years ago)	−0.026 （0.047）
2012 (2 years ago)	−0.042 （0.035）
2014	0.059 （0.045）
2015 (1 year later)	0.172* （0.095）
2016 (2 years later)	0.281** （0.120）
2018 (4 years later)	0.285* （0.146）
2019 (5 years later)	0.313** （0.137）
Constant	11.718* （6.619）
Control variables	Yes
Individual fixed effect	Yes
Time fixed effect	Yes
Observations	390
$R^{2}$	0.963

Note: * * *, * * and * denote statistical significance at the levels of 1%, 5%, and 10%, respectively. The brackets are robust standard errors, and the standard errors are clustered at the provincial level.

**Fig. 4:**
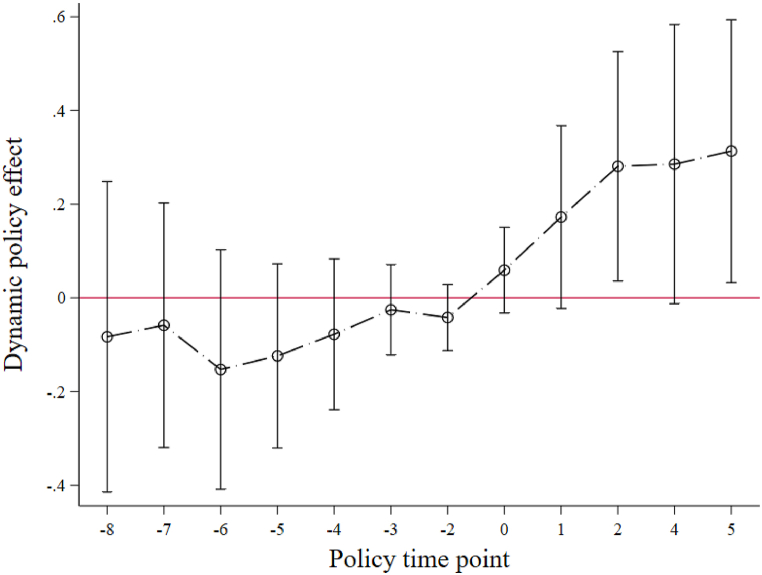
Dynamic effect of carbon market policy.

To more intuitively show the impact of carbon market policy on the low-carbon development of the power industry and the dynamic trend of the policy effect, this paper draws the dynamic effect diagram of carbon market policy (Fig. 4). Among them, the negative sign "-" indicates before the policy was implemented, "0″ indicates 2014 (the year when the policy was implemented), and the positive number indicates after the policy was implemented. In addition, because 2013 (the year before the implementation of the policy) is the base year and there is a lack of a comprehensive score in 2017, Fig. 4 does not show the policy effects in 2013 and 2017.

### 5.2 Propensity score matching-related test

To alleviate the differences between groups that do not change with time and the possible non-randomness problems in the establishment of pilot areas, this paper further uses the PSM-DID model for analysis. To ensure matching quality, propensity score matching needs to pass the common support test and the balance test.

(1) Common support test

**Fig. 5:**
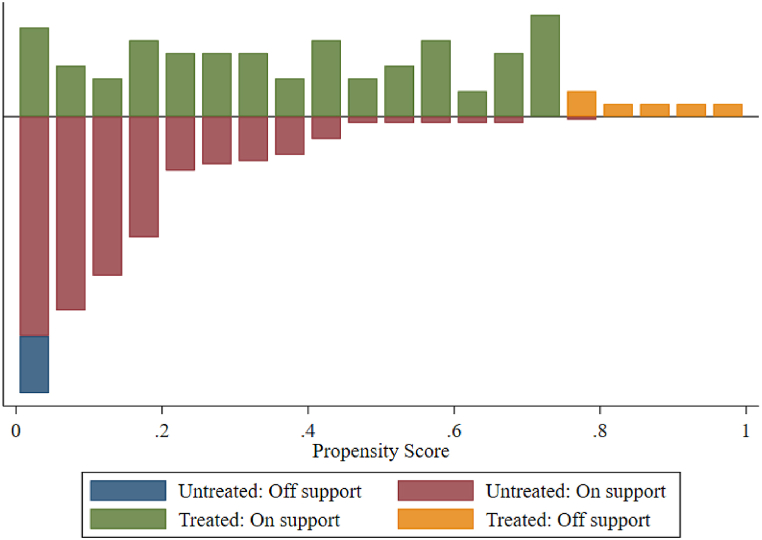
Common support area.

With the treatment variable (${treat}_{i}$) as the dependent variable and the economic development level (GDP), industrial structure (IS), population size (PS) and environmental regulation (EGI) as the corresponding matching variables, logit model regression is carried out, and the common supporting areas are obtained, as shown in Table 9. Among the 390 samples, 366 samples are on support, which indicates that the propensity scores of the treatment group and the control group are mostly in the common range, but only a few samples are off support. Therefore, it passed the common support test. To show the common support area more intuitively, Fig. 5 is drawn.

(2) Balance test

**Table 9:** Common support inspection.

Group	Common support		Amount to
	Off support	On support	
Control group	18	294	312
Treatment group	6	72	78
Amount to	24	366	390

**Fig. 6:**
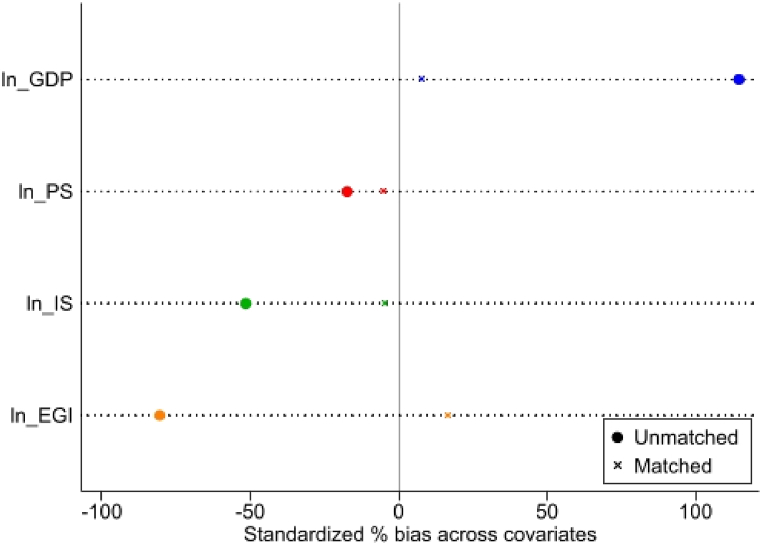
Covariate standard deviation before and after matching.

As shown in Table 10, the absolute value of the standard deviation of covariates ranges from 4.5% to 16.7% after nearest neighbor matching with calipers, and the absolute value of the standard deviation of most covariates is less than 10%. The absolute values of the standard deviations of covariates are obviously smaller than those before matching, and the decreasing range is 69.0%–93.2%. In addition, all covariates do not reject the original assumption that there is no systematic deviation in the values of the two covariates after matching. Therefore, it passed the balance test. To show the change in the standard deviations of covariates before and after matching more intuitively, Fig. 6 is drawn, where the horizontal axis is the standard deviation and the vertical axis is the covariate.

**Table 10:** Balance test.

Variable	Sample	Mean value		% Standard deviation	% Standard deviation reduction degree	T test	
		Treatment group	Control group			T-statistics	P value
lnGDP	Front matching	10.949	10.315	114.5	93.2	9.17	0.000
	After matching	10.881	10.837	7.8		0.47	0.636
lnIS	Front matching	−0.967	−0.831	−51.5	91.2	−4.91	0.000
	After matching	−0.925	−0.913	−4.5		−0.26	0.795
lnPS	Front matching	8.081	8.208	−17.4	69.0	−1.35	0.177
	After matching	8.109	8.149	−5.4		−0.30	0.767
lnEGI	Front matching	−7.368	−6.651	−80.5	79.3	−6.81	0.000
	After matching	−7.230	−7.379	16.7		0.99	0.326

**Fig. 7:**
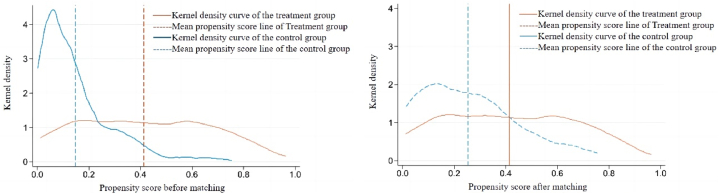
Kernel density distribution and mean value of the propensity score before and after matching.

Fig. 7 shows the kernel density distribution and mean value of the propensity score before and after matching. After matching, the deviation of the kernel density curve between the treatment group and the control group is greatly reduced, the probability density of propensity score values is relatively close, and the difference between the treatment group and the control group is reduced from 0.266 to 0.161. The matching effect is good.

### 5.3 The PSM-DID model result

To avoid the influence of individual differences and time effects on the comprehensive score of the low-carbon development of the power industry, and through the Hausman test, the models from column (2) to column (5) in Table 11 have carried out two-way control of individual and time fixed effects. Column (1) indicates the results of the ordinary DID model; Column (2) shows the DID model results of the two-way fixed effect; Column (3) indicates the PSM-DID model results using samples with nonnull weights; Column (4) indicates the PSM-DID model results using samples satisfying the common support hypothesis; Column (5) indicates the PSM-DID model results using frequency weighted regression.

**Table 11:** Benchmark regression results.

Variable	DID		PSM-DID		
	Ordinary	Two-way fixed effects	The weight is not empty.	Common support	Frequency weighting
	(1)	(2)	(3)	(4)	(5)
Treat*post	0.228**	0.275***	0.253**	0.275***	0.257**
	(0.096)	(0.091)	（0.114）	（0.096）	（0.111）
Constant	−9.087***	10.624*	8.977	9.999	10.712*
	(2.455)	(6.142)	(7.003)	(6.145)	(6.076)
Control variables	Yes	Yes	Yes	Yes	Yes
Individual fixed effect	Yes	Yes	Yes	Yes	Yes
Time fixed effect	No	Yes	Yes	Yes	Yes
Observations	390	390	139	366	203
$R^{2}$	0.824	0.956	0.962	0.955	0.971

Note: * * *, * * and * denote statistical significance at the levels of 1%, 5%, and 10%, respectively. The brackets are robust standard errors, and the standard errors are clustered at the provincial level.

The results show that the coefficients of interaction term (treat*post) between grouped dummy variables and dummy variables before and after the implementation of the policy are positive in both the abovementioned DID model and the PSM-DID model, and the interaction term coefficients of the models from column (1) to column (5) are significant at 5%, 1%, 5%, 1% and 5%, respectively, which indicates that the carbon market policy has an obvious promoting effect on the low-carbon development of the power industry.

In the PSM-DID model, the interaction term coefficients of samples with nonnull weights, common support hypothesis samples and frequency weighted regression are 0.253, 0.275 and 0.257, respectively, and the results of the three are close. Considering that whether the samples with nonnull weights or the samples satisfying the common support hypothesis are used for regression, one problem is neglected: the matched control group samples may be the matching objects of multiple treatment groups, so the importance of control group samples with different weights in the overall control group samples is different. The greater the weight is, the more times it is matched, and more attention should be given to participating in the regression. Therefore, this paper finally adopts the result of frequency weighted regression, and the implementation of carbon market policy makes the comprehensive score of low-carbon development of the power industry in pilot areas increase by 0.257 points.

### 5.4 Robustness test

This paper adopts the following two methods to test the robustness: (1) Adjust the sample period. Limited by data, the sample period of this paper is 2006–2019 (excluding 2017). Considering that environmental policies such as green certificates and energy use rights trading started to be eliminated in 2017, to exclude the influence of other environmental policies as much as possible, this paper shortens the time window and adjusts the sample period to 2006–2016. (2) Winsorize the sample. To alleviate the influence of extreme values in variables on the robustness of regression results, this paper carries out bilateral winsorization on the 1% quantile.

The results of the robustness test are shown in Table 12, in which the models in column (2) and column (4) control the individual and time fixed effects. The results show that after adjusting the sample period or winsorizing the sample, the regression coefficient and significance of treat*post are consistent with the DID results in Table 11 above, indicating that the benchmark regression results in this paper are robust, and the carbon market policy has a positive effect on the low-carbon development of the power industry.

**Table 12:** Robustness test results.

Variable	Adjust the sample period		Winsorize the sample	
	Ordinary	Two-way fixed effects	Ordinary	Two-way fixed effects
	(1)	(2)	(3)	(4)
Treat*post	0.210**	0.220***	0.220**	0.245***
	(0.088)	(0.080)	（0.093）	（0.088）
Constant	−9.663***	11.504	−9.327***	9.442
	(2.695)	(7.304)	(2.467)	(5.900)
Control variables	Yes	Yes	Yes	Yes
Individual fixed effect	Yes	Yes	Yes	Yes
Time fixed effect	No	Yes	No	Yes
Observations	330	330	390	390
$R^{2}$	0.798	0.953	0.825	0.956

Note: * * *, * * and * denote statistical significance at the levels of 1%, 5%, and 10%, respectively. The brackets are robust standard errors, and the standard errors are clustered at the provincial level.

## 6 Conclusions and policy implications

Based on the broad concept of "low carbon", this paper constructs a low-carbon development evaluation index system of China's power industry with 20 specific indicators in five subsystems of carbon emissions, pollution emissions, energy utilization and consumption, economic benefits and social benefits. The analysis model calculates the "comprehensive score for low-carbon development of the power industry" to describe the low-carbon development level of the power industry in 30 provinces in China. Then, the kernel density estimation method is used to analyze the dynamic change trend and characteristics of the low-carbon development level of the power industry at the national level and the three major regional levels, and the characteristics of the low-carbon development level of the power industry at the provincial level are described. Then, the spatial distribution characteristics of the low-carbon development level of China's power industry are analyzed using the K-means clustering method. On this basis, the propensity score matching double difference method (PSM-DID) is used to study the impact of carbon market policies on the low-carbon development level of China's power industry. The specific conclusions are as follows:

(1) The low-carbon development level of China's power industry is generally improving, but the dynamic changes in the low-carbon development level of the power industry between regions and provinces show different characteristics. The specific performance is that the low-carbon development level of the power industry in the country and the three major regions of the east, central and west has been significantly improved, but the growth rates of each region and within the region are different, and there are obvious differences. From the perspective of the whole country, the absolute difference in the low-carbon development level of the power industry in various provinces in China is showing a trend of narrowing, but at the same time, it is showing a trend of polarization; from the perspective of the three major regions, the low-carbon development level of the power industry among the three major regions is different. However, this difference has gradually decreased over time. The growth rate of the low-carbon development level of the power industry in the central and western regions was significantly higher than that in the eastern region. The gap in the level of development shows a trend of widening, while the gap in the level of low-carbon development of the power industry in the provinces in the central region and the western region shows a trend of large and small narrowing, respectively.
(2) The low-carbon development level of China's power industry has regional effects and gradient effects, and there are obvious spatial differences. The specific performance is as follows: Looking at the country as a whole, the low-carbon development level of the power industry presents a trend of gradually decreasing from east to west. From high to low, the low-carbon development level of the power industry is in the eastern region, the central region and the western region. The provinces of the region are mainly distributed in the third and fourth tiers. From the perspective of echelon composition, the echelons to which most provinces belong have not changed, and the spatial distribution of the low-carbon development level of the power industry is relatively stable, but at the same time, it shows the characteristics of agglomeration from lower-level echelons to higher-level echelons. Compared with the third and fourth echelon provinces, the first and second echelon provinces have higher economic levels and superior resource endowments, and most of the carbon market policy pilot provinces are in the first and second echelon.
(3) The carbon market policy has a positive effect on the improvement of the low-carbon development level of China's power industry. The empirical results show that the carbon market policy has significantly improved the low-carbon development level of the power industry in the pilot areas. Moreover, the policy effect of the carbon market has the characteristics of time lag and growth. One year after the pilot of the carbon market started, the policy effect began to appear. With the passage of time, the positive impact of the carbon market policy on the low-carbon development of the power industry gradually expanded.

Based on this, this paper obtains the following policy implications:

(1) The low-carbon construction of the power industry should include five levels: carbon emissions, pollution emissions, energy utilization and consumption, economic benefits and social benefits. When planning the low-carbon transformation path of the power industry and designing related low-carbon policies, we should not only focus on the most direct purpose—to reduce carbon emissions—but also comprehensively consider the other four levels to maximize the low-carbon transformation of the power industry. The comprehensive benefits of carbon development can improve the development quality of the power industry.
(2) Excavate the driving factors and deep-seated reasons that cause the differences in the low-carbon development level of the power industry in different provinces and construct the low-carbon transformation path of the power industry according to local conditions. This paper mainly explores the influence of policy factors on the low-carbon development of the power industry. Local governments should fully consider the influence and mechanism of environmental policies on the low-carbon development of the power industry, and under the unified layout of national carbon market policies, they should put forward corresponding supplementary policies according to regional characteristics to maximize the positive effects of environmental policies.
(3) Establish the linkage mechanism of low-carbon development between regions and provinces and promote the coordinated development of the low-carbon transformation of the power industry in different regions. Provinces with a low level of low-carbon development in the power industry should actively learn from the experience of provinces with a high level or great progress and strengthen cooperation and exchanges at the technical and institutional levels. Considering that the overall regional differences are made up of regional differences and provincial differences, the government should further improve the top-level design of the low-carbon development of the power industry, make overall plans from the national and regional levels, give a certain policy inclination and support to the regions with low-carbon transformation levels of the power industry in the central and western regions, supplement regional shortcomings with policy advantages, and narrow the gap in the low-carbon development level of the power industry among regions and provinces to realize the low-carbon development of the power industry as a whole and promote the early realization of China's double-carbon goal.
(4) Accelerate the construction of the national carbon market and increase the carbon market participation of the power industry in each province. The results show that regional carbon market policies can significantly promote the low-carbon development of the power industry. Therefore, under the circumstances that the low-carbon development of the power industry in the carbon market pilot areas has achieved results, the incentive mechanism of the national carbon market should be further improved, the enthusiasm for carbon trading in the power industry in various regions should be increased, and more power companies should be included in the national carbon market. In addition, provinces with noncarbon trading pilots before the official launch of the national carbon market should take more effective measures to promote the local power industry to adapt to and integrate into the national carbon market system as soon as possible to give full play to the role of the carbon market in promoting the low-carbon development of the power industry.

There are still some deficiencies in the research of this paper. First, due to the different statistical calibers of the data, there may be some deviations between the obtained comprehensive scoring results and the actual ones. In addition, this article takes China's data as an example to analyze whether the abovementioned low-carbon development evaluation index system of the power industry is applicable to other countries and regions, which deserves further consideration and exploration. Follow-up research can make some adjustments to the indicators of the low-carbon development evaluation index system of the power industry in this paper to adapt to the actual situation of the corresponding countries and regions.

## Declarations

### Author contributions statement

Hao Liang: Conceived and designed the experiments ;Wrote the paper. Yingying Zeng: Performed the experiments. Xuchu Jiang: Analyzed and interpreted the data ;Wrote the paper. Ying Li: Contributed reagents, materials, analysis tools or data.

### Funding statement

The research is supported by Hubei Province Education Science Planning Project (2021GB010) , and by Philosophy and Social Sciences Research Project of Hubei Provincial Department of Education (21G032).

### Data availability statement

Data associated with this study has been deposited at:

https://libvpn.zuel.edu.cn/s/www.epsnet.com.cn/index.html#/Index

https://libvpn.zuel.edu.cn/s/www.cnki.net/

https://www.ceads.net.cn/

### Declaration of interests statement

The authors declare that they have no known competing financial interests or personal relationships that could have appeared to influence the work reported in this paper.

### Additional information

No additional information is available for this paper.
